# Motion sensations, postural sway, and side effects for copolar galvanic vestibular stimulation

**DOI:** 10.1007/s00221-026-07255-4

**Published:** 2026-02-28

**Authors:** Caroline R. Austin, Luc Willett, Torin K. Clark

**Affiliations:** University of Colorado Boulder, Smead Aerospace Sciences, Boulder, CO USA

**Keywords:** Tilt perception, Balance, Montage, Bipolar, Pitch, Skin tingling

## Abstract

Galvanic vestibular stimulation (GVS), traditionally applied with oppositely polarized electrodes on the two mastoids (behind the ears), produces self-tilt/rotation perceptions and postural reflexes primarily about the head-centered roll axis (i.e., out the nose). However, there are reports of non-vestibular side effects, such as skin tingling at the electrodes, metallic taste, and visual flashes, which have not been systematically characterized. Previous work suggests pitch sensations may be produced by introducing a distal electrode(s) and co-polarizing the mastoid electrodes, but copolar montages have not been compared. In this series of experiments investigating GVS for creating pitch sensations, we first quantified the relative prevalence, strength, and directionality of sensations induced by 2 previously used copolar electrode montages, along with the typical binaural bipolar montage. Subjects were blinded to montage in all studies. Across current amplitude and waveform (DC and various sine waves), we found both copolar montages produce noticeable motion sensations in roughly half of participants, predominantly in the anterior-posterior (pitch) direction and corresponding postural sway, but substantial side effects, particularly for skin tingling. To compare four copolar montages, we performed force-choice experiments, with pairings of different GVS montage and stimuli. Copolar montages all produced similar effects, including anterior-posterior sway, but the distal electrode on the Forehead tended to induce greater motion sensations, and the Shoulder had less side effects, for equal current applied at the mastoids. In sum, this work systematically characterizes motion sensations, postural sway responses, and the side effects associated with various copolar montages and stimulus parameters.

**Supplementary Information:**

The online version contains supplementary material available at 10.1007/s00221-026-07255-4.

## Introduction

Galvanic vestibular stimulation (GVS) is a technology capable of changing vestibular processes that has been known about for 2 centuries (BOS and JONGKEES 1963), but is of growing interest for applied use. GVS artificially hyperpolarizes (anode) or depolarizes (cathode) the vestibular organs through electrodes traditionally placed on the skin over the mastoid processes (i.e., behind the ears). The change in polarization caused by the electrical stimulation results in a decreased or increased firing rate (respectively) of the vestibular neurons, ultimately affecting vestibular-mediated reflexive and perceptual processes (Fitzpatrick and Day 2004; Forbes et al. 2023). It has been widely shown that hyperpolarizing one set of vestibular organs (e.g., in the right ear) while depolarizing the other (e.g., in the left ear, or vice versa), a configuration predominantly known as “binaural bipolar”, causes responses in the head-centered roll plane (with some small yaw contributions) (Njiokiktjien and Folkerts 1971; Coats 1973a; Séverac Cauquil et al. 2000; Wardman et al. 2003; Fitzpatrick and Day 2004; Day et al. 2010; Ehtemam et al. 2012; Aoyama et al. 2015; Niehof et al. 2019; Aedo-Jury et al. 2020). There has also been some investigation into hyperpolarizing or depolarizing both sets of vestibular organs at the same time; to be comprehensive we refer to this configuration as “copolar” in this paper, though previous papers have termed these electrode montages as binaural monopolar(Cauquil et al. 1998; Day et al. 2011), monopolar bi-aural/binaural (Njiokiktjien and Folkerts 1971; Hlavacka and Njiokiktjien 1985; Magnusson et al. 1990), bilateral monopolar (Aedo-Jury et al. 2020), double monaural (Séverac Cauquil et al. 2000), bilateral unipolar (Fitzpatrick and Day 2004), double fronto-mastoidal (Cauquil et al. 1998), and same directional anteroposterior stimulation (Aoyama et al. 2015). Copolar montages have been shown to induce responses in the head-centered pitch plane, though typically the response is weaker than that observed in the lateral direction for binaural bipolar.

Given the ability of GVS to alter vestibular signals, it has been considered for a variety of commercial and training applications. Multiple groups have investigated GVS to increase presence and reduce cybersickness in VR (Cevette et al. 2012; Sra et al. 2019; Groth et al. 2022). Others have investigated using GVS to recreate or counteract piloting illusions such as “the leans” (Kim et al. 2023), post roll illusion (Houben et al. 2024), somatogravic illusion, and Coriolis cross-coupling illusion (Pradhan et al. 2022). Additionally, GVS has been investigated as a means for mimicking astronaut post-flight vestibular deficits (Moore et al. 2006; Moudy et al. 2024) or as a countermeasure to motion sickness (Allred et al. 2025). All of these applications will benefit from a more detailed characterization and quantification of perceptual responses to GVS. Moreover, characterizing the side effects alongside the desired responses will help researchers and developers make more informed decisions about the best parameters to select for their desired applications.

Previous efforts have started characterizing the vestibular effects of GVS. Indeed, the effects of the binaural bipolar montage have been well studied. (Njiokiktjien and Folkerts 1971) began characterizing typical sway response curves using a 4s 1.5 mA direct current (DC) stimulus where unrestrained participants sway initially towards the cathode followed by a larger sway towards the anode and others have confirmed this response with different current amplitudes and stimulus times, regardless of electrode placement (Séverac Cauquil et al. 2000) and head position (Lund and Broberg 1983; Day et al. 2010). Sinusoidal sway responses at a variety of different frequencies (0.025–5.2 Hz) and current amplitudes (0.05–2 mA) have also been characterized, finding that GVS increases the sway at the frequency of application and that larger sway responses occur at lower sinusoidal frequencies and larger current amplitudes (Coats 1972; Hlavacka and Njiokiktjien 1985; Latt et al. 2003; Ehtemam et al. 2012; Simoneau et al. 2025). In addition to vestibular-mediated postural responses to GVS, others have investigated resulting perceptions of self-motion. (Wardman et al. 2003) showed that the perceptual responses are typically in the same direction of sway (towards the anode) when participants are unrestrained, but in the opposite direction of the anticipated sway response (towards the cathode) when participants’ heads are fixed. More recent work has quantified the effect binaural bipolar GVS on roll perception. (Niehof et al. 2019) measured the effect of up to 2 mA of DC stimulation on subjective visual vertical with the subject seated upright; (Gallagher et al. 2023) measured supine rotational perceptions to +/-1 and 2 mA DC stimuli through a physical motion cancelling paradigm; and (Allred et al. 2024) measured the effect of multiple sum of sines waveforms of up to 4 mA on subjective haptic horizontal during corresponding sum of sines physical tilt motions, resulting in a model capable of predicting self-tilt perception for an arbitrary physical motion and bipolar GVS stimulus. Other vestibular mediated reflexes such as muscle activation for balance control, and VOR have also been studied (Karlberg et al. 2000; Fitzpatrick and Day 2004; Ghanim et al. 2009). Some studies mention additional side effects due to GVS including tingling or pain of the skin at the electrode site (Cevette et al. 2012; Tax et al. 2013; Sra et al. 2019; Groth et al. 2022; Houben et al. 2024) or a metallic taste in the mouth (Tax et al. 2013). In addition, we have had participants mention that in some cases they experienced visual flashes (phosphenes) when GVS current was applied as has (Nissi et al. 2024). To the best of our knowledge, these side effects have only been recorded anecdotally and never systematically tracked and reported.

Sway and perceptual responses have also been investigated in copolar montages to a lesser extent and with varying electrode placements. In copolar montages, electrodes are also placed on each mastoid, and additional “distal” electrode(s) (sometimes called “indifferent” electrodes) are placed elsewhere on the head or body to allow a complete circuit where the mastoid electrodes are the same polarity and the distal electrode(s) are of opposite polarity. (Njiokiktjien and Folkerts 1971) commented that copolar GVS caused an anterior posterior (AP) disturbance and anodal mastoids resulted in backwards sway, without further quantification. (Magnusson et al. 1990) found similar results while working to confirm this AP response was vestibular in nature using a neck based distal electrode and (Cauquil et al. 1998) characterized the sway displacement and latency to DC stimuli ranging from 0.4 to 1 mA with distal electrodes on the forehead. Responses to a 0.3 Hz sinusoidal waveform were investigated by (Hlavacka and Njiokiktjien 1985) using distal electrodes on the hands. Considering perception, (Cevette et al. 2012) provides initial quantification using a single forehead electrode; 20s +/-1.5-2.5mA DC stimuli; and a joystick reporting task. (Aoyama et al. 2015) measured both objective sway and subjective forced choice perceptual responses to 3 mA DC stimuli to show that a copolar montage with distal electrodes placed on the temples produced sway and perceptions consistent with previous work, but the authors suggest that their electrode placement may be superior based upon their modeling of current pathways in the head. Work by (Séverac Cauquil et al. 2000), however, showed the equivalence of binaural GVS postural responses (both bipolar and copolar) to the sum of two monoaural stimuli responses using 4s 0.6 mA DC stimuli, suggesting that sway responses are primarily mediated by the polarization of the vestibular system. Together, these efforts show that multiple copolar montages can produce anterior-posterior postural and perceptual responses, but *do not* investigate the same range of frequencies and current amplitudes as used for the binaural bipolar montage, compare copolar montages, or assess side effects.

In this study, we first attempted to characterize the magnitude, direction, and timing of perceptual and sway responses as well as the severity and prevalence of side effects induced by two previously used copolar montages at a variety of current amplitudes and frequencies. We compared them directly with each other and the binaural biopolar montage to determine if one copolar montage produced stronger motion sensations or weaker side effects in blinded participants. Finding the copolar montages to be comparable, we investigated two additional montages, again with the goal of maximizing pitch perceptual and sway responses while minimizing uncomfortable side effects. Following this second evaluation, we concluded that in general all montages produced similar outcomes with slight differences in motion sensations and side effect tolerability.

## Methods

Thirty participants provided written informed consent under Protocol 22–0171, approved by the Institutional Review Board at the University of Colorado Boulder. Each sub-experiment protocol had 10 participants. All participants were between 18 and 65 with no known conditions that would impact balance or vestibular function. For all experiments, participants were outfitted with 2 × 2in sponge electrodes soaked in 8 ml of saline in the appropriate locations for that experiment’s montages. Before placing electrodes, the skin was prepped with alcohol swabs and NuPrep (Weaver and Company) skin exfoliant. Electrodes were secured with headbands and tape as appropriate. Impedances were confirmed to be below 12kΩ between all electrode pairs for all subjects.

**Fig. 1:**
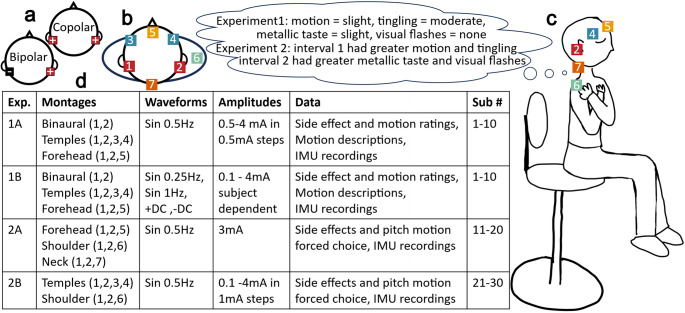
Four sub experiments were performed using 5 different GVS montages to characterize motion sensations, sway, and side effects as well as compare copolar montages. **a** Diagram indicating positive sign convention for bipolar and copolar GVS such that the positive anode is on the right mastoid **b** Top view illustration of electrode locations. **c** Side view illustration of electrode locations and participant positioning (eyes closed, arms crossed, seated upright with back and feet unsupported) during experimental trials. Participants were instructed to pay attention to motion sensations and side effects during trials and made reports at the end of the stimulation. **d** Table summary of the 4 sub experiments including information on the montages/electrode locations used, GVS stimulation properties, the types of data collected, and which subjects participated

Five electrode montages were used across experiments (Fig. 1). First is the “Binaural” montage where an electrode is placed on each mastoid and current is directed in a bipolar manner, such that one electrode is a cathode (-) and the other is an anode (+). All other montages are copolar; include the two mastoid electrodes; and the name describes the location(s) of the distal electrode(s) where plural names indicate that there are two symmetrically placed electrodes at that body location (i.e., Forehead, Temples, Neck, and Shoulder). The current in these copolar montages is directed such that both mastoid electrodes have the same polarity (i.e., both anodes or both cathodes) while the distal electrode(s) have the opposite polarity of those placed at the mastoids.

GVS current waveforms were applied via an in-house device which allowed for up to 5 independent electrodes, enabling switching between montages without unplugging or relocating electrodes during an experiment. When discussing stimulus amplitude, we typically refer to the maximum current applied at any single electrode, thus, for the 3 electrode montages (Forehead, Neck, Shoulder) only half the amount of listed current was being applied at each mastoid (noted by listing the mastoid current in parentheses, when applicable).

### Experiment 1

We began by directly comparing two montages previously found to induce pitch perceptions, Forehead (Cevette et al. 2012) and Temples (Aoyama et al. 2015), as well as the Binaural montage, in 10 participants (24.2 ± 3.4 years, 6 F). During trials, participants were instructed to close their eyes in the well lit room and sit relaxed with their feet off the ground, head straight and upright, back straight and away from the chair back, and arms folded across the chest in order to limit visual and somatosensory cues of orientation (Fig. 1c). After assuming the described position, participants received a 12s GVS waveform, after which they were asked to verbally rate their sensations of motion, tingling on the skin, metallic taste, and visual flashes, each on a scale of none, slight, moderate, severe where “slight” was defined as at least noticeable and “severe” was defined as intolerable. The experimenters described what each of the sensations/side effects might feel like (i.e. a sensation of feeling pushed/pulled or otherwise moving; a prickling/tickling/burning sensation on the skin at the electrode site; a taste of pennies or blood in the mouth; and a bright flash(es) across the eyes while closed), but participants received no additional training on how to detect sensations or rate the magnitude and we re-iterated that participants may or may not experience any of these sensations throughout the experiment or on any given trial. For reports of motion, participants were asked to describe the motion type (tilt/translation/general instability); direction (roll/pitch/yaw/left/right/forward/back/up/down/circular); timing (rhythmic/continuous static/continuous dynamic/intermittent); and any other description they felt was relevant (often comparisons to other real-life motion experiences or details explaining a more complex perception). Participants were provided with a note card reminder of the questions and suggested descriptors.

A head mounted IMU (MBIENTLab MetamotionRL) recorded postural sway at 25 Hz for only 6 of the 10 participants due to equipment malfunctions. IMU data was processed using the Matlab imufilter.m function with the corresponding sampling frequency to compute Euler angles for use in generating rotation matrices to align the vertical axis of the IMU with gravity and the other two axes with the anterior-posterior and medial-lateral axes (Lonner et al. 2023). Following alignment, the data was parsed by trial. Roll, pitch, and yaw angles were calculated via integration of the aligned angular velocities with a corrective 0.01 weighted averaging with trigonometric computations using the accelerometer data to reduce drift. These values were plotted for visualization as seen in Fig.S4. For sinusoidal GVS waveforms (see Experiment 1 A and 1B), the power spectral density (PSD, periodogram.m function in Matlab) and sway magnitude ($\:\sqrt{{PSD}_{freq}*2}$) were computed at each stimulus frequency using the full 12s IMU signal and log transformed for statistical analysis. For the DC waveforms, angular sway displacement from the start of GVS to 5s was computed.

### Experiment 1A

The 12s waveform was always a 0.5 Hz sinewave while the montage and current amplitude were manipulated. Here, and for all experiments, subjects were blinded to the GVS presentation (montage, waveform, amplitude). On each trial the montage (Binaural, Forehead, or Temples) was randomly selected, while the current amplitude started at 0.5 mA and was incremented by 0.5 mA (1 mA, 1.5 mA, etc. up to 4 mA) each time a participant reported all side effects as less than severe for that montage. If participants did report severe side effects the amplitude was decreased by 0.5 mA until the participant reported all non-severe (e.g., moderate, slight, or none) side effects again. This took 20–30 trials and coarsely estimated the maximum tolerable current amplitude (high) and the minimum required amplitude to induce motion sensations (low) for each montage, in addition to capturing the intensity of motion sensations and side effects as a function of current amplitude across the 0.5-4 mA range.

### Experiment 1B

Next, the type of waveform was varied, as well as the current amplitude and montage. Four new waveforms were tested: 0.25 Hz sinewave, 1 Hz sinewave, +DC, and –DC where + is defined by the right mastoid as the anode. For each montage (Binaural, Forehead, Temples), we used the maximum tolerable and minimum motion inducing current amplitudes determined in Experiment 1 A. The 0.25 Hz and –DC waveforms were also tested at a 0.1 mA amplitude as a sub-perceptual and postural threshold (Yang et al. 2015; Nissi et al. 2024) reference/control condition for each of the montages. These 30 trials were administered in a random order. This experiment allowed us to assess the sensations and side effects across a range of waveform frequencies likely to be used in future applications.

### Experiment 2

Rather than subjective ratings (none, slight, moderate severe), here the effectiveness of each montage was evaluated through forced choice comparisons over two intervals. This allowed us to more directly determine whether one montage was perceived as better than another. Participants received the same descriptions of sensations/side effects and were positioned as in Experiment 1 (eyes closed, feet off ground, back straight, arms crossed).

Participants received 12s, 0.5 Hz sinusoidal waveforms during both intervals, but the montage used differed in each interval, such that each montage was tested against the others (e.g., Forehead vs. Neck or Shoulder), in a series of randomized counterbalanced presentations. After the two intervals, participants made forced choice judgements (i.e., that from the first interval or second) on the strength of pitch (forward/backward) motion sensations and side effects (tingling, metallic taste, and visual flashes). We counted selections across trials to quantify the effectiveness of each montage (for the pitch motion sensations and each side effect, separately). Forced choice comparisons were evaluated against the null 50% selection rate indicating equal severity motion sensations and side effects. Additionally, an IMU (XSENS Movella Dot) secured to the back of the subject’s head recorded anterior-posterior sway at 30 Hz for comparison between the montages and against the subject’s reports. We processed the data as in Experiment 1 and again quantified sway PSD and magnitude at 0.5 Hz (now for the middle 10s of the 12s IMU recording to exclude edge effects in these recordings), employing a log transform of the median for each condition to meet parametric assumptions.

### Experiment 2A

In our 2 A cohort (*N* = 10, 22 ± 3.5 years, 8 F), we tested the Forehead, Shoulder, and Neck montages. Using only montages with 3 electrodes enabled direct comparison of more montages (given the 5 electrode constraint of our system) and ensured that the current applied at both the mastoids and distal electrode would be the same for each montage, reducing variability from Experiment 1. The Neck montage was selected for its use in (Magnusson et al. 1990). The Shoulder montage was selected after internal piloting suggested that placing the electrode(s) on top of the shoulder had more tolerable skin tingling than the head. Each montage was tested against the others at an amplitude of 3(1.5 at the mastoids)mA four times for each pairing.

### Experiment 2B

In our final cohort of 10 participants (25.6 ± 3.9 years, 6 F), we tested the Temples and Shoulder montages. We selected the Shoulder montage from Experiment 2 A because it appeared to have weaker side effects than the Forehead montage, which we had already compared to the Temples montage in Experiment 1. We wanted to directly compare the montages across different current amplitudes. Noting that the current amplitude applied to the mastoids in the Shoulder montage is half the amplitude applied to the mastoids for the Temples montage (for a given maximum current), we evaluated how the current applied at the mastoid versus the maximum current applied at any electrode effected pitch sensations and side effect severity. The Temples montage was tested at maximum amplitudes of 0.1 (sub perceptual threshold), 1, 2, 3, and 4 mA while the Shoulder montage was tested at maximum amplitudes of 0.1, 2, 3, and 4 mA (0.05, 1, 1.5, 2 at mastoids). The 0.1 mA amplitudes were tested against the maximum amplitude for the same montage, but all other (above threshold) conditions were tested only against the other montage conditions in all possible pairings. Each pairing was tested twice to allow for counterbalancing.

## Results

### Experiment 1A verbal reports

**Fig. 2:**
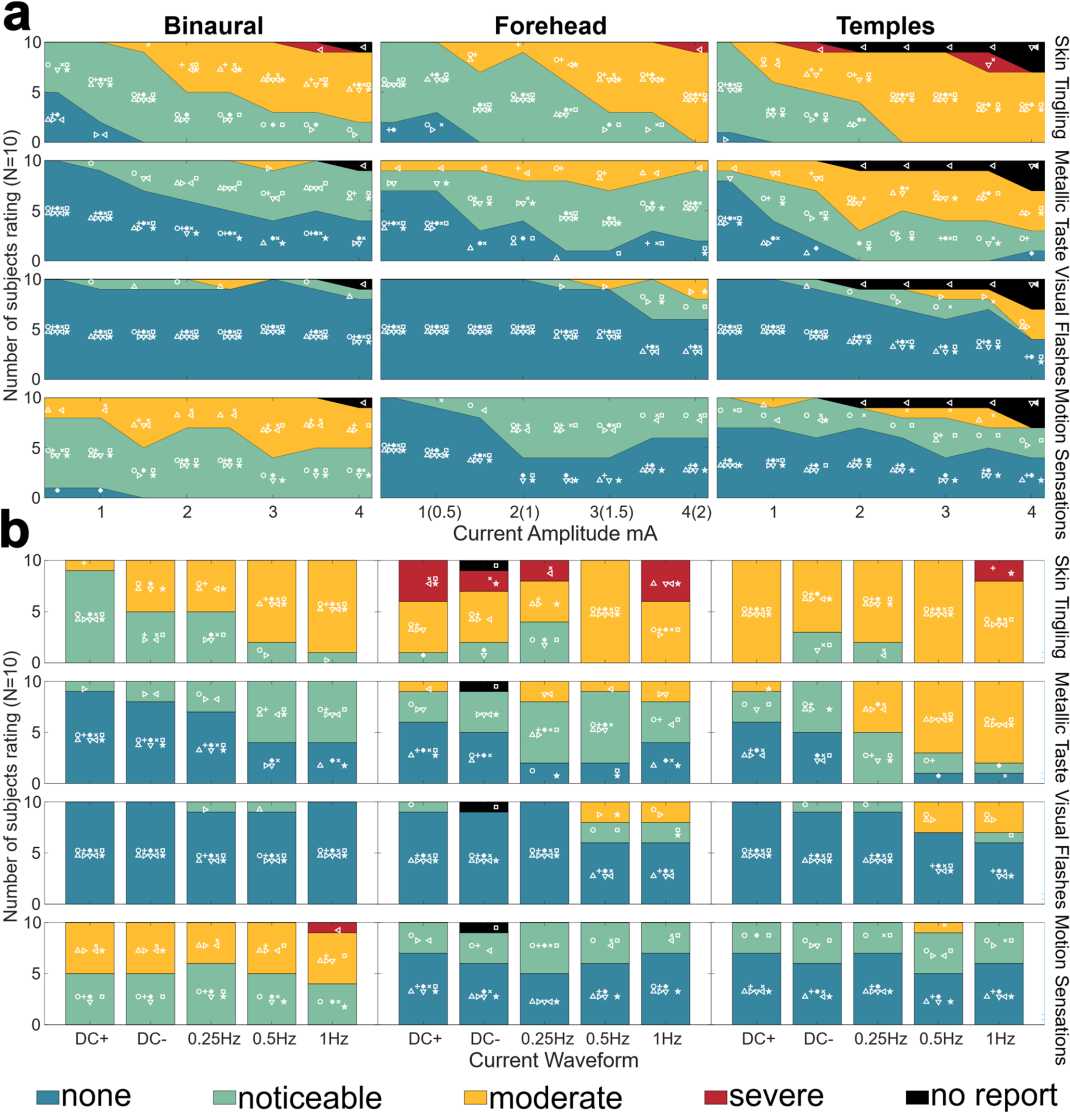
Participants experienced presentations of 12s GVS waveforms with either the Binaural (left column), Forehead (center), or Temples (right) montages at maximum current amplitudes of 0.5-4 mA. After each presentation, participants then rated side effects (skin tingling, metallic taste, visual flashes) and motion sensations (top to bottom) on a scale of none, slight, moderate, or severe. The total number of ratings are shown through the color coded area in the plot and a unique symbol for each of the 10 participants is plotted in the color region corresponding to their report, enabling tracking individual participants across conditions. **a** Results for a fixed GVS waveform of 0.5 Hz sinusoid and current varying from 0.5 to 4 mA. Report severity tended to increase with increasing current amplitude. **b** Results with the maximum tolerable current amplitude determined from part A (for each subject and montage), now across different GVS waveforms. Some side effects tended to be worse for sinusoidal/higher frequency waveforms, but motion sensation intensity was consistent across waveforms

The number of subjects reporting motion sensation and side effects (skin tingling, metallic taste, and visual flashes) as a function of GVS current amplitude (0.5 -4 mA) is shown in Fig. 2a, for the Binaural, Forehead, and Temples montages. Severity of skin tingling increased with increasing current amplitude, as expected, and all three montages generated strong tingling sensations. The Temples appeared to generate the strongest tingling sensations, with all subjects reporting feeling at least a moderate severity at 2.5 mA and higher. Across the experiment, three participants reported severe sensations of tingling.

Metallic taste reports were also strongest for the Temples, followed by the Forehead and Binaural. All participants reported at least a noticeable metallic taste for the Forehead and Temples while 2 of the 10 participants never reported a sense of metallic taste for Binaural. There were no severe reports of metallic taste.

Visual Flashes were the least prevalent side effect with only 2 out of 10 participants reporting visual flashes for Binaural, and 4 out 10 for both Forehead and Temples. Visual flashes appeared to be stronger at higher current amplitudes for the Forehead and Temples, particularly at 3 mA and above.

Motion sensations were the most prevalent and intense with Binaural. All 10 participants reported at least noticeable sensations across the 0.5-4 mA range and 6 out of 10 reported moderate sensations of motion. For Forehead, 8 out of 10 participants reported noticeable sensations of motion at least once across the 0.5-4 mA range, but none reported moderate. For Temples, 8 out of 10 participants reported noticeable sensations of motion at least once and 2 out of 10 reported moderate sensations of motion. Across Temples and Forehead montages, even at higher amplitudes up to 4 mA, roughly half of participants reported “none” motion sensations. There was some consistency in which subjects were less susceptible to motion sensations across montages: the participant (inverted triangle symbol) that reported “none” for Forehead and Temples, as well as one (star) that reported “none” for Forehead and one (asterisk) for Temples, each also never reported moderate for Binaural.

**Fig. 3:**
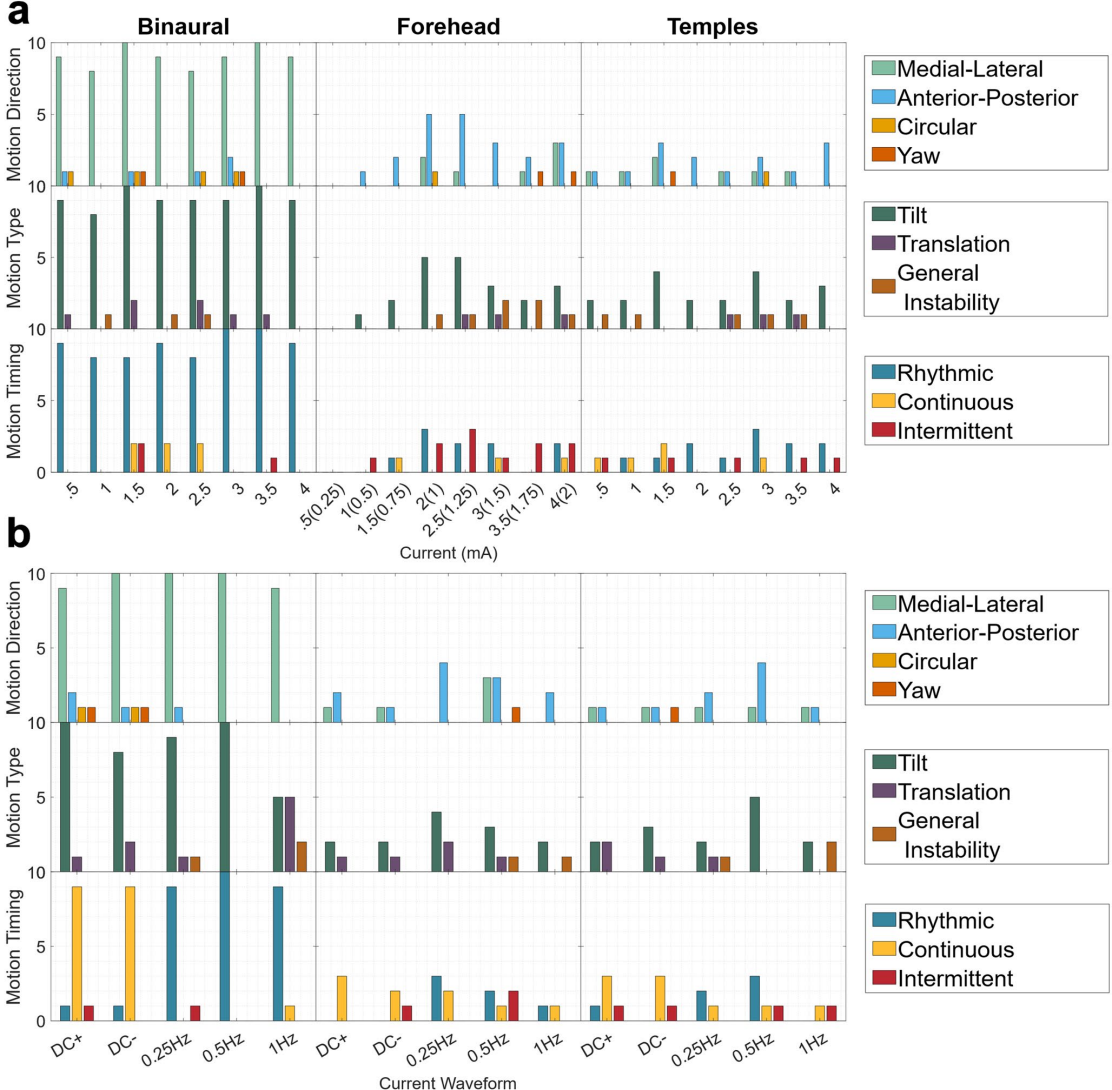
Participants experienced presentations of 12s GVS waveforms with either the Binaural, Forehead, or Temples montages at maximum current amplitudes of 0.5-4 mA. After each presentation if a participant reported a motion sensation associated with a stimulus they were asked to describe the motion direction, type, and timing. Participant responses were coded based on key word descriptors (participants responses could use multiple key words) and the number of participants that used each descriptor is shown by the colored bars. **a** Shows the results from a fixed 0.5 Hz GVS waveform and varying current amplitude. Binaural resulted in the most sensations of motion which were mostly of rhythmic medial lateral tilt. Forehead and Temples had fewer reports but were mainly of intermittent/rhythmic anterior posterior tilt. **b** Shows the results from varying waveforms at the maximum tolerable current amplitude determined in Experiment 1 A. Again, the Binaural montage had more reports across waveforms of mainly medial lateral tilt sensations (although the 1 Hz sinusoid had equal reports of translation). DC stimuli resulted in reports of continuous motion while sinusoids were rhythmic in nature. Forehead and Temples induced mostly sensations of pitch tilt again with DC stimuli being described as more continuous and sinusoidal stimuli being described as rhythmic or intermittent

When motion was reported. its direction, type (tilt, translation, general instability), and timing (rhythmic, continuous, or intermittent) were characterized in Fig. 3a. For Binaural, most motion sensations were in the medial-lateral direction, and sensations of tilt were dominant to sensations of translation. Most participants also reported that sensations induced by the 0.5 Hz waveform were rhythmic. All of these results are consistent with existing literature in which sinusoidal binaural bipolar GVS predominantly induces an oscillatory roll tilt sway (Latt et al. 2003; Ehtemam et al. 2012). This sensation was consistently reported even at the lowest current amplitude of 0.5 mA for Binaural. For Forehead there were fewer reports of motion sensations, but motion direction in the anterior posterior plane was reported most often. Similar to Binaural, tilt sensations were reported more often than translation or general instability. Timing reports indicate that while the sensations were sometimes perceived as rhythmic, they were also often perceived as intermittent, which is consistent with regards to the sensations being weaker and potentially more ambiguous than those produced by Binaural. For Temples, anterior-posterior motions were most frequently reported, although medial lateral motions were reported as well. Like the other two montages tested, tilt was the dominant motion type, and similar to Forehead, timing reports were mixed with rhythmic being most frequently reported.

### Experiment 1B verbal reports

Motion sensation and side effect ratings for all 5 GVS waveforms at each subject’s maximum tolerable current amplitude are shown in Fig. 2b. A Corresponding plot for the “low” GVS amplitude is available in supplementary information (Fig. S2). For the 0.1 mA condition, there were rarely “false positive” reports with 95.8% of presentations rated as “none”.

Consistent with Experiment 1 A (using only the 0.5 Hz sinusoid waveform), side effects were more intense at the higher current amplitudes, now tested with + DC, -DC, 0.25 Hz and 1 Hz waveforms. Across all 3 montages participants reported at least noticeable tingling sensations with more moderate reports associated with Forehead and Temples. There was similar severity of tingling reported across waveforms. Interestingly, across montages the metallic taste reports tended to be stronger for the sinusoidal waveforms than the DC waveforms. The number of reports of visual flashes remained low, although there was an increase in reports for Forehead and Temples with the sinusoidal 0.5 and 1 Hz waveform. For the maximum current amplitude tolerated by each participant, the motion sensation strength was similar across all waveforms. Again, Binaural was stronger (roughly half “moderate” and half “noticeable”) than Forehead or Temples (at least half “none”, only one “moderate”, and the rest “noticeable”).

Next, we characterized the motion sensation direction, type, and timing for different GVS waveforms (the results for only the maximum current amplitude are shown in Fig. 3b). Binaural resulted in dominantly reports of lateral tilt motion sensations. The 1 Hz waveform did result in an equal number of reports of translation though. The timing for sinusoidal waveforms was dominantly rhythmic while DC waveforms resulted in continuous sensations (either continuous tilt offset or continuous tumbling sensations). Forehead and Temples had fewer and more mixed reports of motion direction, though anterior-posterior and tilt were still the most reported. Similar to Binaural, DC waveforms resulted in continuous motion sensations while sinusoidal waveforms resulted in mixed rhythmic and intermittent reports.

### Experiment 1A and 1B IMU recordings

In addition to subjective reports, we also quantified objective, reflexive postural sway during GVS applications, as compared to natural sway in the 0.1 mA application (where we would expect minimal sway due to GVS). Visualization of IMU traces revealed clear sinusoidal patterns in roll and yaw sway for Binaural and pitch for Forehead and Temples that were generally larger with higher current amplitudes for Experiment 1A.

**Fig. 4:**
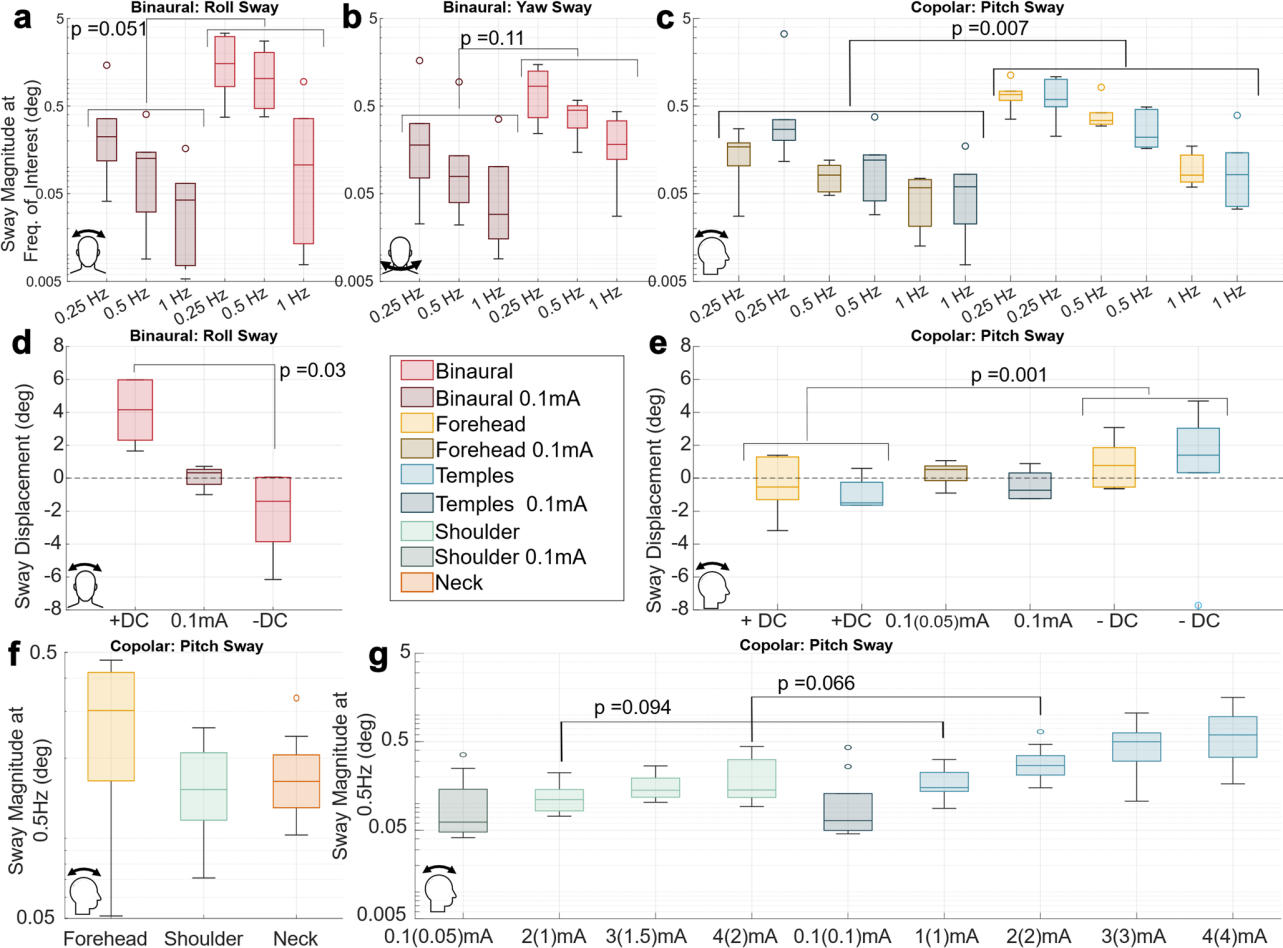
Seated postural sway responses to the 12s GVS stimuli were measured with an IMU mounted on the back of the head. IMU recordings were aligned with the gravitational axis such that the other two axes approximately aligned with anterior-posterior and medial lateral planes. Outcomes are shown only for the direction-montage combinations where the GVS was expected to be most effective. For sinusoidal GVS stimuli the power spectral density (Matlab function periodogram) was used to compute the sway magnitude ($\:\sqrt{{PSD}_{freq}*2}$)). For DC stimuli the displacement from the start of stimulation to 5s was calculated. **a** Compares Binaural sinusoidal sway results in the roll (medial-lateral) plane from the 3 sinusoidal waveforms tested and the DC 0.1 mA condition. There was a significant effect of frequency and a borderline significant effect of GVS. **b** Compares Binaural sinusoidal sway results in the yaw (transverse) plane. Yaw sway showed similar trends to the roll direction, but the GVS effect was not significant. **c S**inusoidal sway results at 0.25,0.5,1 Hz for both copolar montages tested in Experiment 1 (Forehead and Temples). Results between Forehead and Temples were not significantly different and when pooling the data there was a significant effect of GVS application and frequency. **d** Compares DC sway results for the Binaural montage. In the 0.1 mA condition there was little net displacement. Positive (right anode) led to positive (right) sway, negative stimulation led to a negative sway and there was a significant difference between the two conditions. **e** Compares DC sway results for both copolar montages tested in Experiment 1 (Forehead and Temples). There was no significant difference between the Forehead and Temples montages, but there was a significant difference between the positive (mastoids anode) condition which induced negative (backwards) sway and the negative (mastoids cathode) condition which induced forwards sway. **f** Each participants’ median sinusoidal sway for the 0.5 Hz 3 mA waveform tested in the Forehead, Shoulder, and Neck montages; post hoc tests showed no difference between the three copolar montages. **g** Each subjects’ median sinusoidal sway for the Shoulder and Temples montage using a 0.5 Hz waveform at increasing current amplitudes. The peak amplitudes applied (at the distal electrode(s)) are shown with the current applied at the mastoids in parentheses. While the Temples generally had slightly larger sway, post hoc tests between the two sets of conditions with equivalent current applied at the mastoids were not statistically significant

Subsequently we evaluated the sway PSD and magnitude at the frequency which GVS was applied, for the corresponding direction-montage subsets (roll and yaw sway with Binaural and pitch sway with Forehead and Temples) for all sinusoidal waveforms (Fig. 4a-c) For the 0.5 Hz waveform we saw an increase in sway magnitude at 0.5 Hz compared to neighboring frequencies (0–1.25 Hz); other direction-montage combinations within Experiment 1A (i.e., this increase in power at 0.5 Hz was not seen in pitch for Binaural or in roll/yaw for Forehead and Temples); and 0.1 mA/control condition(s) performed in Experiment 1B. Additionally, the magnitude of the 0.5 Hz frequency within the IMU signal tended to be greater for trials with larger GVS amplitudes. Similar trends were seen for the 0.25 Hz and 1 Hz waveform, but qualitative trends were difficult to distinguish for the 1 Hz waveform (data not shown).

To test whether GVS produced more sway in the expected axis at the stimulation frequency than in the DC 0.1 mA presentation, we pooled the log transformed sway magnitude outcomes from all 3 sinusoidal conditions (0.25 Hz, 0.5 Hz, 1 Hz) to perform 3 repeated measures ANOVAs where *p* < 0.05 is considered significant. The first two ANOVAs considered only Binaural and a single axis (one for roll sway amplitude, another for yaw), testing GVS vs. control with a co-variate of the 3 different frequencies. For roll, we found a borderline significant main effect of GVS (F(1,5) = 6.5; *p* = 0.051, Fig. 4a) with less sway at higher frequencies (F(2,10) = 24.8, *p* < 0.001), and no significant interaction. This strong trend in the effect of Binaural GVS (and frequency) on roll sway is consistent with findings from previous studies (Coats 1972; Latt et al. 2003). The yaw sway ANOVA (Fig. 4b) showed only a significant effect of frequency (F(2,10) = 22.2; *p* < 0.001) and not GVS (F(1,5) = 3.87, *p* = 0.11), though GVS trials did trend towards having more sway than with the 0.1 mA control.

The third ANOVA considered only pitch sway for both Forehead and Temples (Fig. 4c), including factors for the two montages, three frequencies, and GVS vs. control. We found a significant effect of GVS and frequency, but no effect of montage (F(1,5) = 0.5, *p* = 0.51) and no interactions. Pooling across the two montages, maintained a significant effect of more sway with GVS (F(1,5) = 19.45; *p* = 0.007) and at lower frequencies (F(2,10) = 82.8; *p* < 0.001).

Finally, we considered sway responses from +/-DC GVS waveforms (Fig. 4d&e). For Binaural, we observed roll sway to the right for + DC and left for –DC, with a non-parametric paired t-test finding a significant difference between the two (*p* = 0.03). The same test was performed for pitch sway, separately for both Forehead (*p* = 0.09) and Temples (*p* = 0.03), with backwards sway for + DC and forwards sway for -DC. Even though Forehead only approached significance, pooling across two montages as before yielded a significant difference in resulting postural pitch sway angle between +/-DC GVS waveforms (*p* = 0.001). These results are consistent with prior findings (Séverac Cauquil et al. 2000), but now with alternative copolar montages.

### Experiment 2A verbal reports

**Fig. 5:**
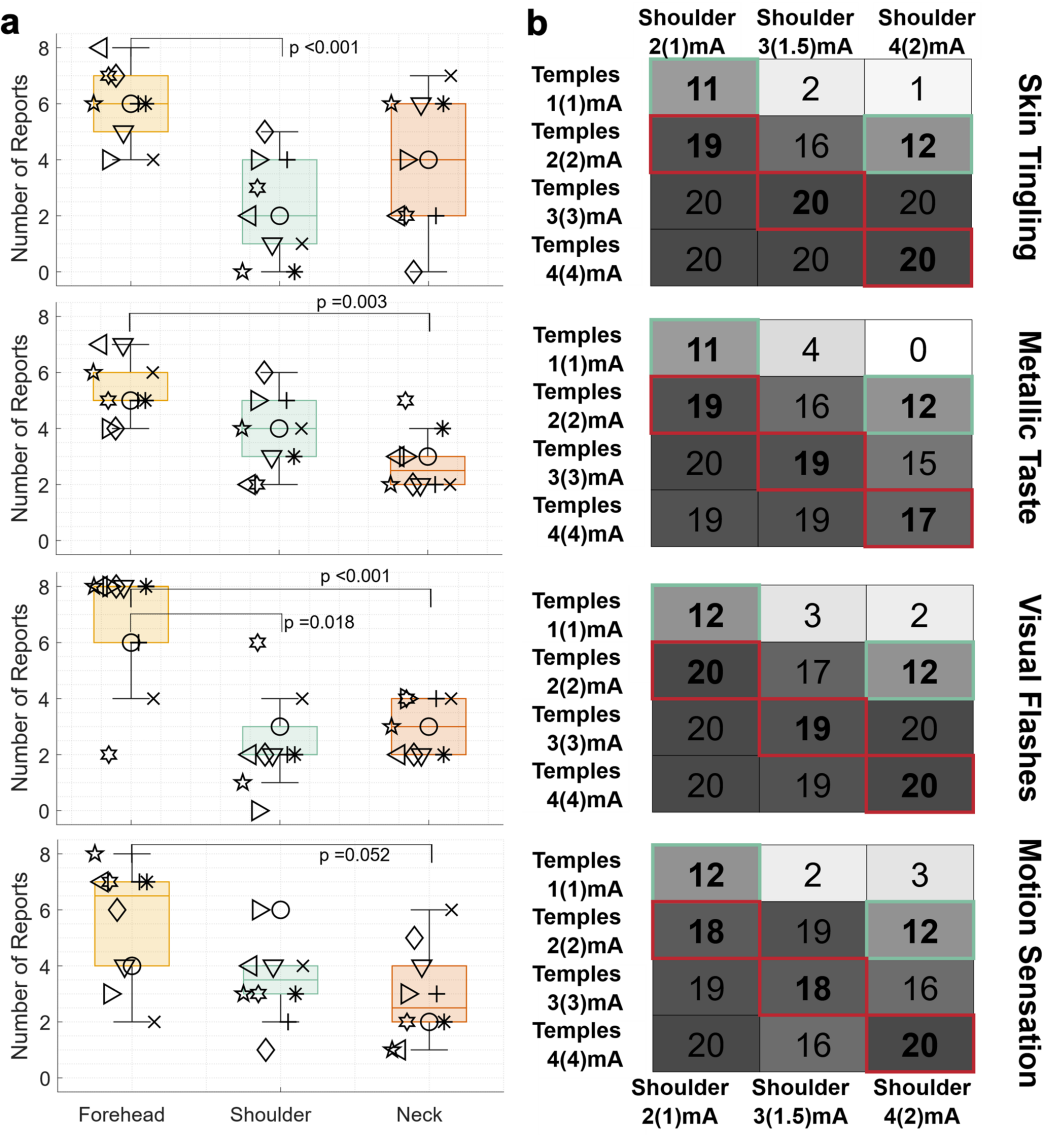
Participants experienced 2 intervals of 12s 0.5 Hz sinusoidal GVS waveforms with different montages and made forced choice comparisons regarding the strength of side effects and forward/backward/pitch motion sensations. **a** Participants compared the Forehead, Shoulder, and Neck montages at 3 mA four times each. The Forehead montage had significantly stronger side effects than at least one of the other two montages for all three side effects considered (skin tingling, metallic taste, visual flashes). The Forehead had borderline significantly stronger motion sensations compared to the Neck montage. **b** Participants compared the Shoulder and Temples montages at amplitudes from 1-4 mA at the distal electrodes (and the mastoids shown in parenthesis) The value in each square indicates the number of times the Temples montage condition (labeled on the left) was selected as stronger than the Shoulder montage condition (label on top and bottom). The squares with green outlines indicate conditions with equal current applied at the mastoid; red outlines indicate conditions with equal maximum current applied at any electrode. For all side effects and motion sensations the results in the green squares are not significantly different than chance ratings, but squares above and to the right are significantly different than chance such that the Shoulder montage was stronger and squares below and to the left are different than chance such that the Temples montage was stronger

To reduce variability from subjective rating (“none”, “slight”, etc.), we asked for forced choice decisions of intensity for pitch motion sensations and side effects (skin tingling, metallic taste, visual flashes) following consecutive interval pairings of different GVS montages (which were summed across comparisons to quantify montage efficacy for Forehead, Shoulder, and Neck, see Fig. 5a). There were large inter-individual differences and no unanimously preferred montage across subjects. We performed a one sample t-test against the 50% pure chance probability (i.e., if all montages produced equal effects) for each montage pairing and sensation/side effect. The responses for skin tingling were significantly non-equal for Forehead vs. Shoulder (*p* < 0.001), with Shoulder being less intense. For metallic taste, Neck was less intense than Forehead (*p* = 0.003). Both Shoulder and Neck had less intense visual flashes compared to Forehead (*p* = 0.018,*p* < 0.001). For motion sensations, Forehead was marginally significantly stronger than Neck (*p* = 0.052).

### Experiment 2A IMU recordings

Visualization of pitch data revealed sinusoidal sway, during 0.5 Hz GVS for Forehead, Shoulder, and Neck. The sway PSD and magnitude were evaluated at the GVS frequency of 0.5 Hz (see Fig. 4f). The log transformed median sway magnitude was used in a repeated measures ANOVA comparing montages. There was a significant difference in sway magnitude between the three montages (F(2,18) = 5.3 *p* = 0.016), but post hoc paired t-tests with Bonferroni corrections were not significant between any conditions. We confirmed that sway magnitude was greater in pitch than roll (p < < 0.001) by pooling data across montages in a pairwise t-test.

### Experiment 2B verbal reports

Next, the Temples and Shoulder montages were evaluated in forced choice comparisons across pairings of various current amplitudes. As expected, the montage with the greater current amplitude applied at the mastoids was consistently reported to induce stronger motion sensations and side effects. For example, of the 20 paired presentations (2 per subject for counterbalanced ordering, 10 subjects), the Temples montage with 3 mA was reported to produce a stronger motion sensation than Shoulder with 2 mA (1 mA at the mastoids) 19 times. Using a binomial test for each pairing (each cell in Fig. 5b), we found a significant difference (*p* < 0.05) from 50% pure chance in all pairings except conditions where the current applied at the mastoids was equal (highlighted in green). This aligns with our hypothesis that motion sensation would be primarily a function of mastoidal current and not a function of distal electrode location. We also hypothesized that side effect severity would primarily be a function of the maximum current applied (equivalent cells highlighted in red), but these results suggest that either side effects are also primarily a function of mastoidal current or that the distal electrode location is an important co-factor where Shoulder produces less severe side effects compared to Temples. The sub-threshold comparisons (0.1 mA Shoulder/Temples vs. 4 mA Shoulder/Temples) were excluded from Fig. 5b, but in all cases the 4 mA waveform was rated stronger than the 0.1 mA waveform.

### Experiment 2B IMU recordings

Visualization of the Shoulder and Temples data revealed the same sinusoidal sway patterns in pitch that generally increased with increasing current amplitude. The sway PSD and magnitude were evaluated at 0.5 Hz for each trial (Fig. 4g) showing the same increase in sway power with increasing current amplitude within a montage. Shoulder appears to produce less sway in pitch; a repeated measures ANOVA for conditions with equivalent mastoidal current (Shoulder 2(1)mA/Temples 1 mA, Shoulder 4(2)mA/Temples 2 mA) found significant effects of both montage (F(1,9) = 24, *p* < 0.001) and current amplitude (F(1,9) = 10.6, *p* = 0.01), though follow up paired t-tests, with Bonferroni corrections comparing the montages, did not reach significance in either the 1 mA (*p* = 0.094) or 2 mA (*p* = 0.066) condition.

## Discussion

### GVS postural responses and motion sensations

Our findings regarding the Binaural montage in Experiment 1 were largely consistent with previous work focused on bipolar GVS. Binaural bipolar GVS induced postural responses and sensations of motion at amplitudes as low as 0.5 mA with increasing prevalence and intensity as a function of current amplitude (Fig. 2a). Prevalence and intensity of reports did not appear to vary with waveform (Fig. 2b). Objective sway magnitude was larger for lower sinusoidal frequencies, but this was the case in both GVS and control (DC 0.1 mA) conditions with no interaction (Fig. 4a). Binaural dominantly produced sensations of tilt in the lateral (roll) direction (though 1 Hz sinusoidal stimulation produced equal translation reports). DC stimuli produced mainly continuous sensations of motion, and sinusoidal stimuli produced rhythmic sensations (Fig. 3b). Sway recordings matched these qualitative descriptions (Fig.S4).

While copolar montages are known to induce sway (Cauquil et al. 1998) and motion perceptions(Cevette et al. 2012; Aoyama et al. 2013), primarily in the anterior posterior (pitch) direction, this has not been investigated across a broad range of current amplitudes and GVS waveforms. Additionally, multiple copolar electrode montages have been used, and to our knowledge no group has directly compared responses from different copolar montages to experimentally evaluate the assumed equivalence between montages. Consistent with the results and expectations derived from previous work, we showed that both the Forehead and Temples copolar montages produced sway and motion sensations that were weaker and less prevalent than the sensations experienced in the Binaural montage, but were still achievable in some participants at as low as 0.5 mA with increased prevalence and intensity with increasing current amplitude (Fig. 2a). Sensation prevalence and intensity did not appear to vary as a function of GVS waveform (including sinusoidal frequency, Fig. 2b), though again objective sway intensity measurements did vary as a function of frequency (Fig. 4c&e). Copolar motion sensation descriptions were less consistent between participants, though both Forehead and Temples produced more anterior posterior tilt (pitch) sensations than any other descriptors. DC stimuli produced mainly continuous motion sensations while sinusoidal stimuli were described as rhythmic or intermittent, potentially due to the weakly discernable nature of the sensations (Fig. 3b). While the qualitative aspects of motion sensations were not queried in as much detail during Experiment 2, participants were asked rate montages based only on anterior-posterior motion sensations and informal reports from participants indicated that while the sensations were again often in the anterior-posterior direction, in some trials they perceived motions in alternative directions or no motion at all. Our objective sway data does confirm that participants were experiencing a postural response to GVS with all copolar montages even if it was not reported as perceptually “noticeable” (Fig. 4).

Based on the work of (Séverac Cauquil et al. 2000) we hypothesized that all copolar montages should generate equivalent postural and perceptual responses; however, our results identified some nuances. In Experiment 2 A Forehead tended to induce more intense anterior-posterior motion sensations (*p* = 0.052, Fig. 5a, bottom) and produced objectively larger sway than Shoulder and Neck (F(2,18) = 5.3 *p* = 0.016, but follow up pairwise comparison tests: for Forehead vs. Shoulder *p* = 0.17 with Hedge’s g = 0.79, provided to encourage future investigations or meta-analyses upon these results, and for Forehead vs. Neck *p* = 0.44 and g = 0.62, Fig. 4f); in trends that straddled significance. Experiment 2B found near equivalence of Shoulder and Temples in subjective ratings with equivalent mastoidal current (Fig. 5b, bottom), but there trended towards more sway (Fig. 4g) with Temples (*p* = 0.094, g = 0.95 with 1 mA applied at the mastoids, and *p* = 0.066, g = 1.03 with 2 mA). These results suggest that while there may be an effect of montage on perceptual and postural responses, the effect is typically smaller than that which can be produced from the presence of the GVS and varying stimulus parameters (ex. g = 2.45 for Temples 0.1 mA vs. 4 mA). Additionally, the forced choice selection of larger motion sensations on a given trial aligned with the stimuli which produced greater objective sway results more often than chance (75.4% *p* < 0.001 pooled across Experiment 2 A and 2B). Misalignment tended to occur when the difference in objective sway was smaller which may indicate that small objective differences may not matter perceptually. Our experiments were not designed for comparisons of the objective sway across experiments, but indirect comparisons suggest that the sway response is of a similar magnitude for a given mastoidal current level across all copolar montages tested (Forehead, Temples, Shoulder, Neck) and reaffirms that any differences in montage effectiveness are slight.

### GVS side effects

Consistent with frequent mentions in the literature, we found skin tingling was the most prevalent and most severe side effect. Groups using both bipolar or copolar GVS typically report tingling sensations to be mild at 1 mA (Coats 1973a; Cevette et al. 2012; Aedo-Jury et al. 2020), but as the current amplitude increases there is an increased concern for comfort even at 2 mA (Sra et al. 2019), and participants discontinuing participation due to discomfort above 4 mA (Houben et al. 2024). There is also a concern for participants remaining blinded to GVS experimental conditions (Aoyama et al. 2015). Indeed, while each of our experiments kept electrodes applied even when inactive, and participants were not notified about the montage being presented, they likely were not completely blinded to the montages due to the tactile cues experienced once stimulation started. Our results, particularly the first rows of Fig. 2a and b, provide a course but cohesive map of anticipated tingling severity over a range of current amplitudes and waveforms (DC and sinusoidal frequencies) likely to be used to alter perception. We did not find any clear effects of waveform on tingling severity, although other work has suggested that sharper changes in current amplitude may be perceived as more uncomfortable than slower changes (Groth et al. 2022; Kim et al. 2023). The 1 Hz sinusoid had the fastest changing current amplitudes in our experiment (our DC waveforms included a 1s ramp period) and did have the strongest skin tingling ratings at the maximum current amplitude in Experiment 1B across all 3 montages tested, consistent with this hypothesis. Our results also indicate that the level of discomfort due to skin tingling depends on the location of the electrodes, with distal electrode(s) on the front of the head causing more discomfort. Out of the copolar montages tested, Shoulder appears to produce the most tolerable tingling sensations (Fig. 5a). Additionally, when comparing Shoulder and Temples (Fig. 5b), Temples only had less tingling when the Shoulder montage had at least twice the current amplitude at the distal electrode site. Neck was second most tolerable for skin tingling and considering the ratings in Experiment 1, and the Experiment 2 comparisons against the Shoulder montage, we would suggest that the Temples montage may be slightly more tolerable than the Forehead montage, though further testing would be required to confirm this interpretation. Overall these results match intuition that skin tingling should be more tolerable when distal electrodes are placed on less sensitive skin.

Metallic Taste was the second most prevalent side effect in Experiment 1, though it is not frequently mentioned as a side effect in the literature. While acknowledged as unpleasant by our participants, it was never described as severe (intolerable). Unsurprisingly, metallic taste intensity scaled with current amplitude (Fig. 2a). While prevalent across all waveforms, metallic taste may be stronger for sinusoidal than DC stimuli (Fig. 2b). Similar to skin tingling, metallic taste was more prevalent in copolar montages than the Binaural bipolar montage. Ranking the montages by metallic taste severity suggests that Neck was least intense followed by Shoulder, Forehead, and Temples, but severity was only measured to be significantly different between Neck and Forehead.

Though Visual Flashes were noted as a side effect during internal piloting of Forehead and Temples, there were relatively few reports in Experiment 1, and most were for the copolar montages at higher current amplitudes and higher sinusoidal frequencies (Fig. 2). These frequencies are still considered low compared to other types of stimulation applied to the head which can induce visual flashes and where lower frequencies have been found to be less likely to induce visual flashes (Evans et al. 2019), and so even the inconsistent reports collected here may be surprising despite the high current amplitude and Forehead/Temples location of the distal electrodes. The association with electrode location and sinusoidal waveforms at high current amplitude stimuli may explain why this side effect has not been mentioned in other studies, because to our knowledge this is the first study to investigate copolar GVS with these combined stimulus parameters. We also note that we directly queried participants about visual flashes which has also not been done in previous GVS studies. When forced to choose between montages (Experiment 2), Shoulder and Neck were selected as having less visual flashes than Forehead (*p* < 0.018).

### Selecting a montage

Our results do not indicate a clear “best” choice montage for future use, rather they provide an initial survey of the trade space to inform montage selection. These authors see the primary drivers for montage selection as maximizing motion sensations and minimizing discomfort (primarily due to tingling, though metallic taste may be important for some). While the results suggest that the Forehead montage may produce the strongest sensations, that was not the case for all subjects, and Forehead also produced the strongest side effects. Adding a second electrode to the forehead (“Foreheads”), could further strengthen pitch sensations, but may also exacerbate the side effects. On the other hand, the Shoulder montage had more tolerable side effects, but potentially weaker motion sensations. Again, motion sensations could potentially be increased by adding a second shoulder electrode ("Shoulders"). For studies with limited maximum GVS current amplitudes and short stimulus durations, Forehead(s) or Temples may be a better choice to maximize sensations with moderate side effects. Conversely studies opting to use higher current amplitudes or longer durations may benefit from using a Shoulder(s) montage (or another less sensitive skin location) to minimize discomfort while achieving similar effect sizes from the vestibular driven responses. Other logistical considerations might include hardware setup- it may be easier to use forehead electrodes for an integrated GVS-VR device or a single GVS headband design(Suzuki et al. 2016; Sra et al. 2019; Pradhan et al. 2022; Groth et al. 2022), but fitting electrodes under an existing VR headset may be difficult and uncomfortable. Additionally, electrodes on the forehead/temples had a tendency to slowly slip down the face when secured with just headbands, and both the electrodes and their cables for our setup often occluded the participant’s vision. From this perspective the Neck(s) or Shoulder(s) montages may be better for early prototyping or experiments where participants need to see/move around. Regardless of the montage selected, the additional information about anticipated side effects that this work provides will enable participants to make more informed decisions about the risks of participating in GVS research. Based on our findings we expect that other research groups will continue to use various different montages, and we acknowledge that it is convenient to maintain the same group specific naming conventions; however, we encourage the unified use of “copolar” or “binaural copolar” with a description of the location of the distal electrode(s) in future investigations employing this family of montages.

### Limitations

In this study we utilized two different psychophysical reporting tasks, each with their own limitations. The open ended response nature of Experiment 1 allowed us to capture detailed descriptions of motion sensations, but still required us to distill these descriptions such that a description like “I felt like I was swaying forward but not backward, like there was a slight increasing forward tilt with each pulse” was reduced into the coding of slight (rating), forward (direction), tilt (type), intermittent (timing). In the list of suggested key words for motion type we did not include rotation (and coded yaw rotation as “tilt”), which may have biased participants away from selecting yaw as a motion direction. Additionally, the rating scheme of none, slight, moderate, or severe is relatively coarse and open to subject interpretation. To overcome these limitations, we implemented a two-interval, forced choice paradigm in Experiment 2; however, this method presumes that participants experienced motion sensations and side effects in at least one of the intervals, which Experiment 1 indicated was not always true.

In order to blind participants as to which montage was going to be applied, we used a GVS device and electrode set ups that did not require us to move or unplug electrodes in between presentations in each experiment. This goal, combined with the limited number of electrode ports on the GVS device, limited the number and types of montages we could test within a single experiment. Despite our best efforts for subjects to remain blinded to each GVS presentation, participants may have been able to distinguish montages, current amplitude, and waveform parameters based on the tingling side effect which was felt in most conditions. It is possible that participants associated certain tingling patterns with motion sensations and other side effects experienced in previous trials which could bias responses. For Experiment 2 particularly, we think it is likely that skin tingling may have influenced the forced choice selections for the visual flashes side effect which was frequently absent and thus we would’ve expected to result in equivalence or near equivalence across all conditions. Some participants also reported skin tingling to be a distraction from the motion perception task which may have influenced subjective reports.

This series of studies prioritized the comparison of multiple copolar montages which, given the constraints of our GVS device, we accomplished through a series of smaller complementary experiments. Our detection of marginally significant differences in vestibular and side effects between montages across experiments suggests that there are some effects of montage, but our study design was unable to fully characterize the differences. Future studies looking to refine the quantification of these differences and provide insight into why they exist may benefit from larger sample sizes (*n* ≥ 24 assuming pairwise comparison, α = 0.05, β = 0.2, and g = 0.62 from Experiment 2 A).

GVS alters perception via a vestibular input, but perception is formed through the integration of multiple sensory systems and expectations of motion driven by past experiences which may limit the perception and intensity of motion sensations during GVS. In an attempt to enhance the perceptions induced by GVS, we reduced other sensory cues of perception. Participants performed trials with eyes closed to remove visual cues; and sat on a padded chair away from the back support with feet unsupported to reduce somatosensory cues. Similarly, postural sway responses to GVS depend on the available sensory input and the challenge of the balance task(Day et al. 1997; Cauquil et al. 1997; Fitzpatrick and Day 2004; MacDougall et al. 2006). Here we selected a seated posture rather than a standing balance task to reduce fatigue which likely resulted in smaller postural responses. Thus, GVS applied in other sensory environments like with congruent visual cues or more reliable somatosensory cues may increase or decrease the intensity of the perceptions induced by GVS and GVS applied in more/less challenging balance control tasks may result in increased/decreased postural responses (and thus perceptions) relative to the findings of this work.

Participants were unrestrained in our experimental paradigm, thus the postural sway response to GVS affects motion sensation perceptions. Motion sensation perceptions with the head restrained are expected to be in the opposite direction(Wardman et al. 2003) and may have different magnitudes. While a clear mapping of how the magnitude of perception might change between head-restrained and unrestrained conditions does not exist for copolar montages, (Austin et al. 2024) began looking at correlations between the strength of restrained and unrestrained perceptions for bipolar GVS.

### Future work

This study provides a solid foundation for continued research and application of both bipolar and copolar GVS. In addition to quantifying motion sensations and side effects due to different copolar montages, our work provides a starting point for investigating inter and intra subject GVS response variability, as observed previously (Coats 1972, 1973b). The effect of copolar GVS varied greatly with some participants reporting quite strong sensations, some reporting very weak sensations, and one reporting none. Different participants had different preferred montages for both motion sensations and side effects and even within participants, preferences sometimes varied between trials, and objective sway responses varied for repeated stimuli in Experiment 2. The variability in objective sway responses increased with larger median sway responses, while incongruent forced choice selections were associated with smaller differences in sway responses between intervals. These differences could be further explored to investigate ways to test for individual GVS susceptibility (Sra et al. 2019; Groth et al. 2022; Austin et al. 2024; Allred et al. 2024) that could result in better tuning of GVS amplitudes to achieve the desired magnitude of response. Determining an individual’s integrated perceptual threshold for detecting a sway response to GVS may also be clinically relevant for fall risk evaluation (Simoneau et al. 2025). More than just characterizing this variability, there is the opportunity to understand the factors that cause it. It has been suggested that variability may be caused by differences in sensory weighting (Fitzpatrick and Day 2004), anatomical geometry (Day et al. 2011), or alternative current pathways (Aoyama et al. 2015).

Needs also remain for additional characterization of copolar GVS. Only one study has attempted to quantify how orientation perception can be altered by copolar GVS (Cevette et al. 2012) and this was only tested with the head unrestrained, which is known to produce perceptions in opposite directions than with the head fixed with bipolar responses (Wardman et al. 2003; Fitzpatrick and Day 2004; Day et al. 2011). Thus, we think additional work to quantify copolar GVS alterations to perception (restrained or unrestrained) may benefit future GVS implementation efforts.

## Supplementary Information

Below is the link to the electronic supplementary material.

Supplementary Material 1

## Data Availability

Raw data is available on OSF: https://osf.io/a25b7/overview? view\_only=10813b25a2cf4022a4dbb862e613c6a5.
