# Supplementary material for: Motion sensations, postural sway, and side effects for copolar galvanic vestibular stimulation
Source: Exp Brain Res. 2026 Feb 28;244(4):56. doi: 10.1007/s00221-026-07255-4 (PMC12950039; doi:10.1007/s00221-026-07255-4)
Supplement: Supplementary file 1 — Supplementary Material 1 [file 221_2026_7255_MOESM1_ESM.pdf]

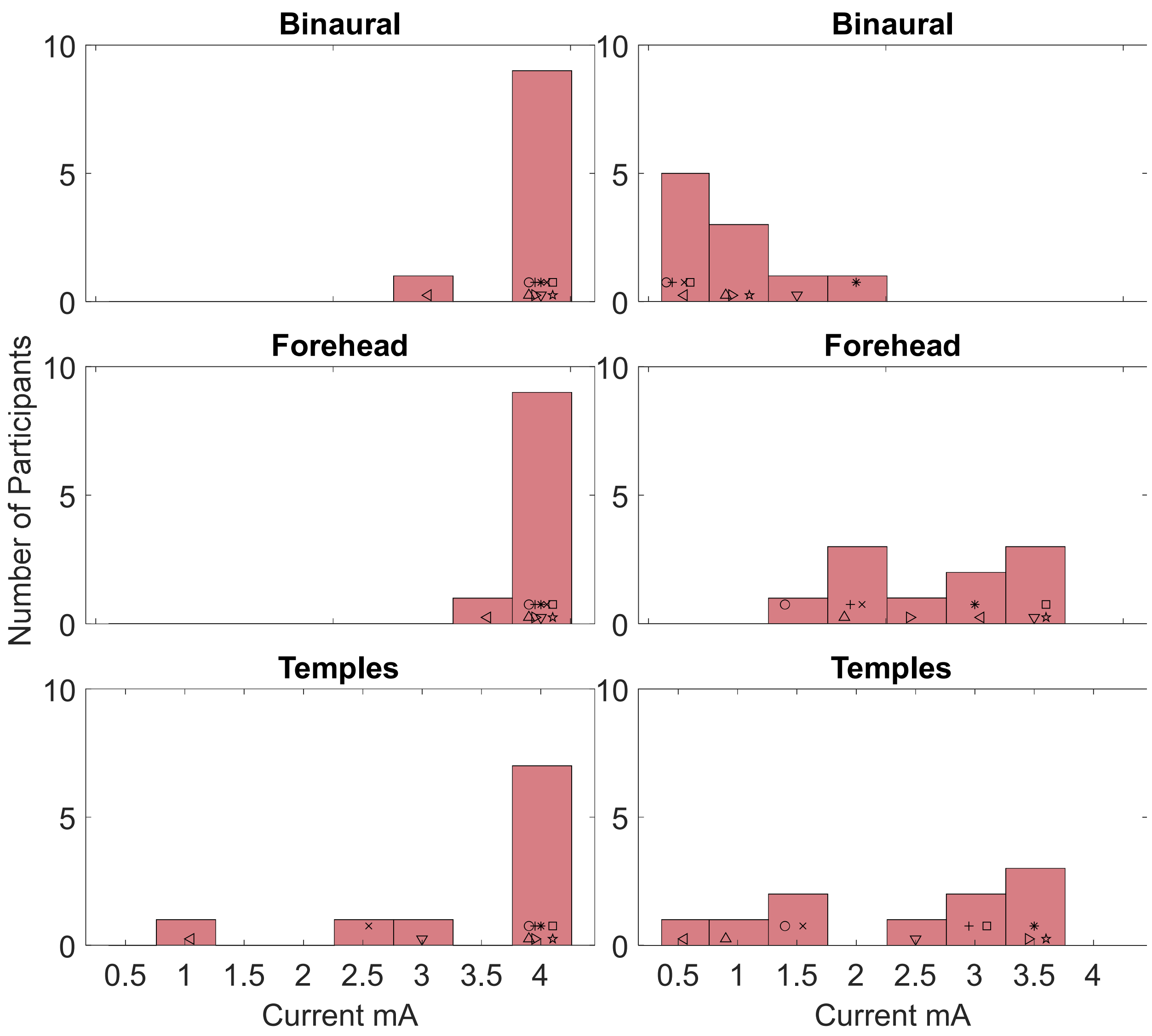

Fig. S1 summarizes the “high” (maximum tolerable, such that all side effects were rated as less than “severe” for that montage) current amplitude (left) and the “low” (at which the participant first reports motion sensation or the second highest tolerable in the case of no motion sensations) current amplitude (right) used in Experiment 1B for each montage and participant based on the results of Experiment 1A. Within the bars of the histogram are symbols for each subject, that are consistent across these panels and the figures in the paper, to track which subjects experienced which “high” and “low” GVS amplitude in Experiment 1B. The maximum possible was 4 mA, with a minimum of 0.5 mA, in 0.5 mA increments. For example, with the Binaural montage (in the upper left), 9 of the 10 subjects tolerated the full 4mA of current amplitude without reporting severe side effects (with the other achieving up to 3mA). For the Binaural montage, in terms of the minimum current necessary for a noticeable motion sensation (upper right) half the subjects felt motion sensations all the way down to 0.5 mA, with three more at 1mA.

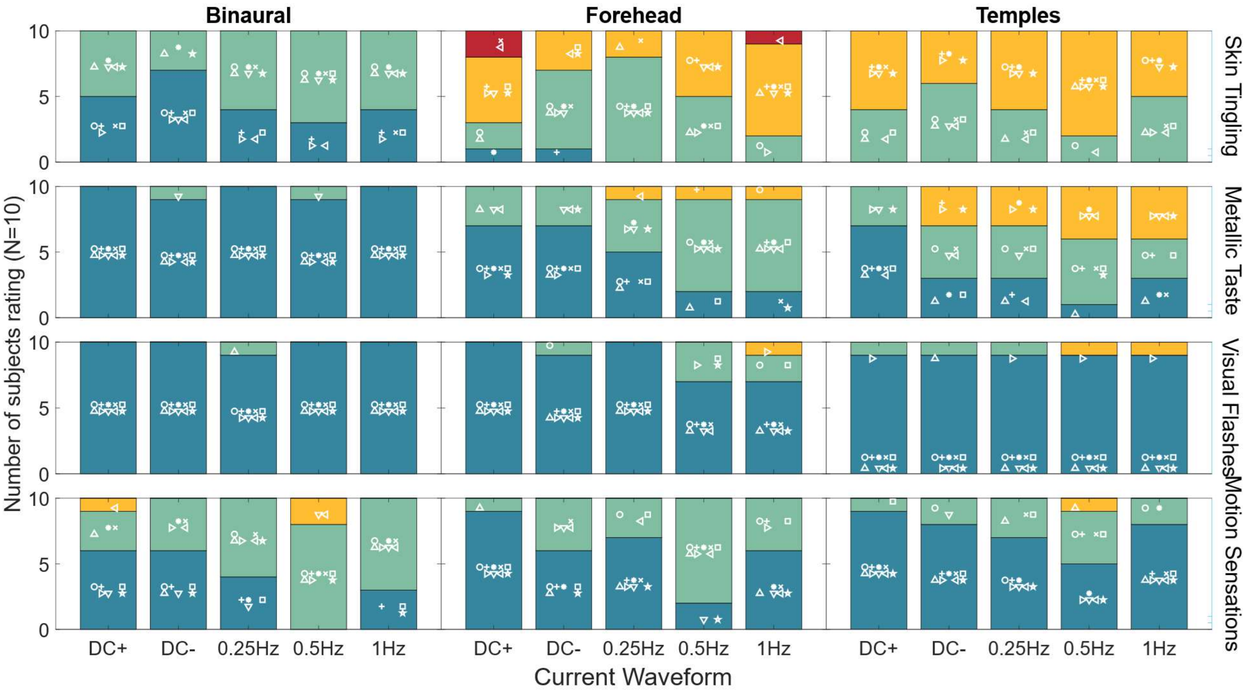

Fig. S2 Results for varying GVS waveforms at the “low” current ampltidue determined from motion sensation reports in Experiment 1A, on a subject and montage specific basis. Participants experienced presentations of 12s GVS waveforms with either the Binaural (left column), Forehead (center), or Temples (right) montages at maximum current amplitudes of 0.5-3.5mA. After each presentation, participants then rated side effects (skin tingling, metallic taste, visual flashes) and motion sensations on a scale of none, slight, moderate, severe. The total number of ratings are shown through the color coded area in the plot and a unique symbol for each of the 10 participants is plotted in the color region corresponding to their report, enabling tracking individual participants across conditions. Reporting trends largely align with the results in Fig.2b which uses the maximum tolerable current, but there is decreased rating severity as expected for a lower current amplitude.

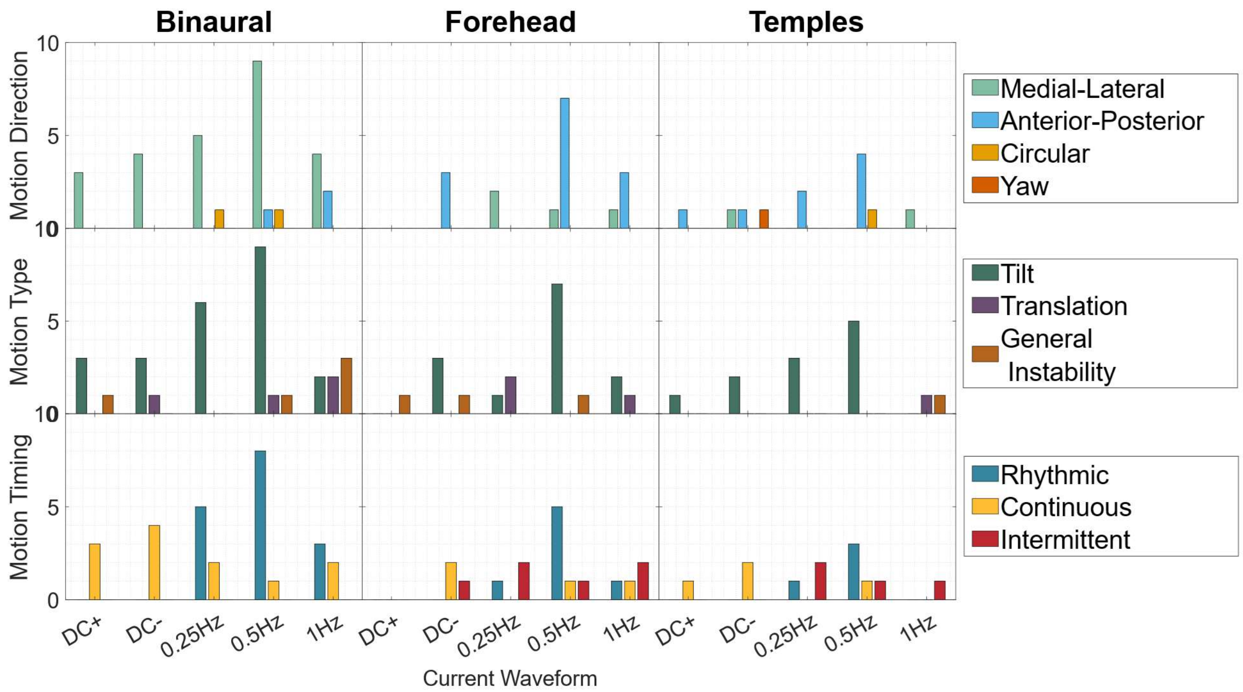

Fig. S3 Results for varying GVS waveforms at the "low" current ampltidue determined from motion sensation reports in Experiment 1A, on a subject and montage specific basis. Participants experienced presentations of 12s GVS waveforms with either the Binaural (left), Forehead (center), or Temples (right) montages at maximum current amplitudes of 0.5-4mA. After each presentation, if a participant reported a motion sensation associated with a stimulus they were asked to describe the motion direction, type, and timing. Participant responses were coded based on key word descriptors (participants responses could use multiple key words) and the number of participants that used each descriptor is shown by the colored bars. Reporting trends align with Fig.3 which uses the maximum tolerable current amplitude.

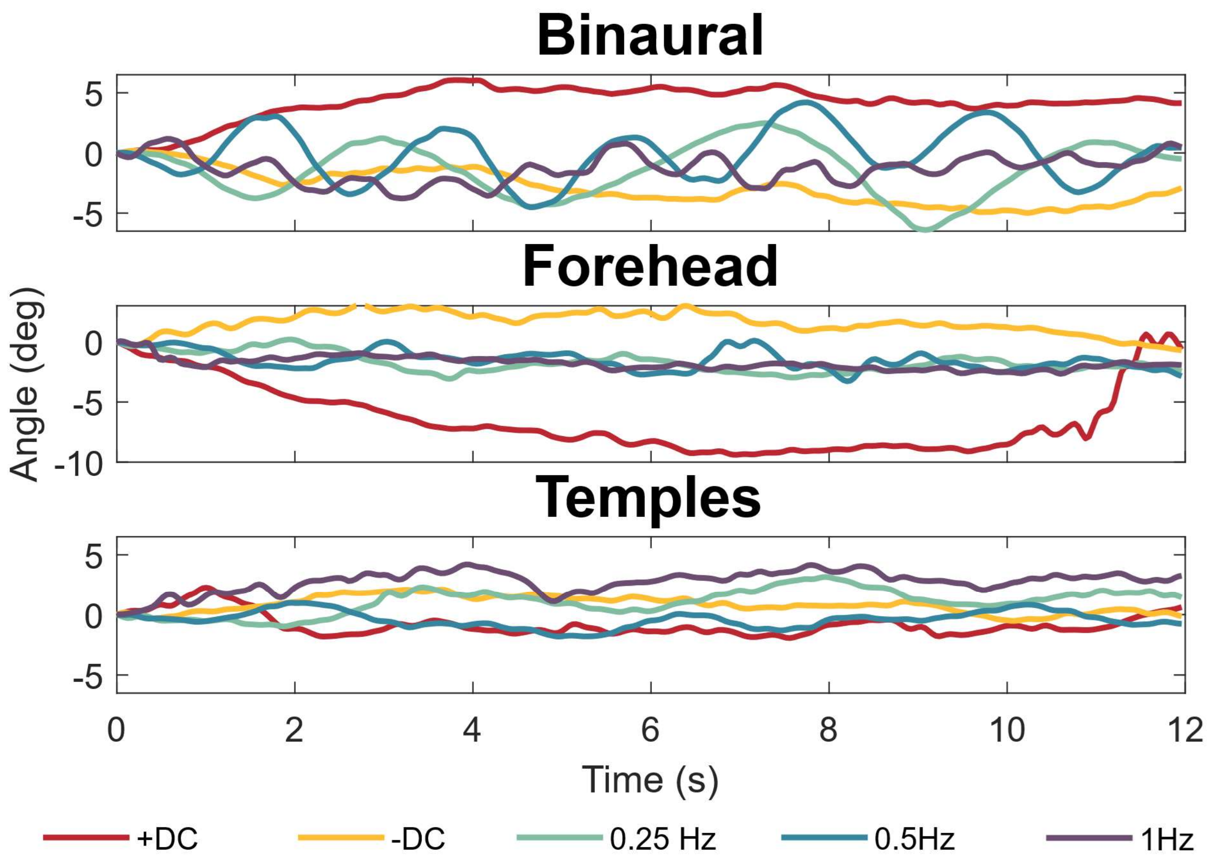

Fig. S4 Example sway responses for a single participant across multiple montages and waveforms in the maximum tolerable current condition. The Binaural (top) plot shows sway in the roll plane while the Forehead and Temples (bottom two) plots show sway in the pitch plane. This participant's sway responses had the most pronounced visual trends. The DC stimuli trace paths in opposite directions and reach a maximum within 4s. For the sinusoidal stimuli a roughly sinusoidal trend at the appropriate frequency can be seen in the data – these trends are clearest in the Binaural (top) plot, but can also be seen in the Forehead and Temples data with smaller amplitudes.
